# Targeting Gut Dysbiosis and Microbiome Metabolites for the Development of Therapeutic Modalities for Neurological Disorders

**DOI:** 10.2174/1570159X20666221003085508

**Published:** 2023-03-27

**Authors:** Matthew D. Wiefels, Emily Furar, Rebecca S. Eshraghi, Jeenu Mittal, Idil Memis, Moeed Moosa, Rahul Mittal, Adrien A. Eshraghi

**Affiliations:** 1 Hearing Research and Communication Disorders Laboratory, Department of Otolaryngology, Miller School of Medicine, University of Miami, Miami, Florida, USA;; 2 Department of Pediatrics, Miller School of Medicine, University of Miami, Miami, Florida, 33136, USA;; 3 Department of Neurological Surgery, Miller School of Medicine, University of Miami, Miami, Florida, USA

**Keywords:** Gut microbiome, gut metabolite, gut dysbiosis, therapeutic modalities, neurological disorders, Alzheimer’s disease, Parkinson’s disease, Autism spectrum disorder

## Abstract

The gut microbiota, composed of numerous species of microbes, works in synergy with the various organ systems in the body to bolster our overall health and well-being. The most well-known function of the gut microbiome is to facilitate the metabolism and absorption of crucial nutrients, such as complex carbohydrates, while also generating vitamins. In addition, the gut microbiome plays a crucial role in regulating the functioning of the central nervous system (CNS). Host genetics, including specific genes and single nucleotide polymorphisms (SNPs), have been implicated in the pathophysiology of neurological disorders, including Parkinson’s disease (PD), Alzheimer’s disease (AD), and autism spectrum disorder (ASD). The gut microbiome dysbiosis also plays a role in the pathogenesis of these neurodegenerative disorders, thus perturbing the gut-brain axis. Overproduction of certain metabolites synthesized by the gut microbiome, such as short-chain fatty acids (SCFAs) and p-cresyl sulfate, are known to interfere with microglial function and trigger misfolding of alpha-synuclein protein, which can build up inside neurons and cause damage. By determining the association of the gut microbiome and its metabolites with various diseases, such as neurological disorders, future research will pave the way for the development of effective preventive and treatment modalities.

## 1 INTRODUCTION

The gut microbiome and the metabolites it produces play an integral role in the regulation of various processes throughout the body, as well as having associations with the pathogenesis of various disorders [1-4]. The microorganisms that inhabit our gastrointestinal (GI) tract play a key role in not only facilitating digestion and nutrient absorption but also regulating the levels of neurotransmitters and hormones, which can affect neurological function and disease progression. For example, metabolites generated by abnormal gut microbiota have been associated with the pathogenesis of certain neurodegenerative disorders, such as Parkinson’s disease (PD) [5-7].

In a healthy state, the gut microbiome consists of over 1,000 microbial species [8, 9]. While these microbe species mainly belong to the phyla *Bacteroidetes* and *Firmicutes*, the gut microbiome has a significant amount of species diversity [10, 11]. The variability that exists between the gut microbiota of different “healthy” individuals also suggests that there may not be a singular microbiome state that is considered healthy, but rather various states, which consist of different compositions of good microbes [11-13]. Studies have suggested that the microbiome of each individual is typically stable over the years but prone to changes and disturbance at very young and elder ages.

**Fig. (1):**
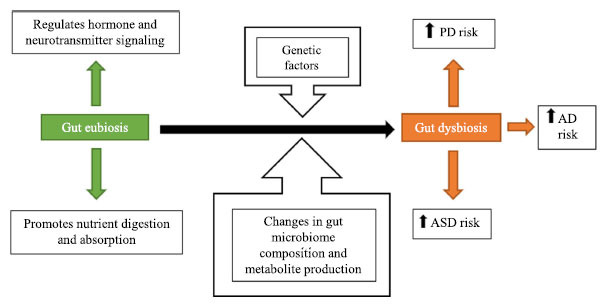
A graphical representation highlighting the role of gut dysbiosis, microbiome metabolites and host genetics in predisposition to neurological disorders, such as Parkinson’s disease (PD), Alzheimer’s disease (AD), and autism spectrum disorder (ASD).

Environmental factors, such as diet and exercise, are known to impact the gut microbiome composition and metabolite profile, which can contribute to the health and disease of an individual [11, 13-15]. Studies have suggested that microbiome diversity may also impact individual phenotypic responses to diet and lifestyle choices. There is continuous research involving the use of gut microbial composition, microbial taxa abundance, and metabolite profile to serve as biomarkers for the diagnosis of various disorders. However, some areas of the relationship between gut dysbiosis and the pathogenesis of diseases remain unclear. Questions, including the importance of microbial alterations in the disease process as well as differentiating between the microbiome composition initiating a disease state for the host *versus* the disease creating variations within the microbiota, remain predominantly unanswered [11]. The objective of this review article is to discuss our current understanding regarding the role of gut dysbiosis and microbiome metabolites in predisposition to neurological disorders, such as PD, Alzheimer’s disease (AD), and autism spectrum disorder (ASD). We also discuss the role of host genetics in altering the gut microbial composition, which can also increase the susceptibility to developing neurological disorders (Fig. **1**). In addition, we highlight the interaction of the gut microbiome with host immune cells, which can lead to neuronal cell damage and apoptosis. A better understanding of gut dysbiosis and microbiome metabolites will provide a catalogue of candidates that can be targeted to develop effective therapeutic strategies for neurological disorders.

## 2 HOST GENETICS AND GUT MICROBIOME

The pathogenesis of several neurodegenerative disorders, including PD and AD, can be due to the accumulation of misfolded or aberrant neural proteins coded for by our own genes. The impact of these genes combined with the host genetics that promotes the dysbiosis of the gut microbiome suggests the significant contribution of genetic factors to the development of these diseases, which hamper the normal functioning of the central nervous system.

Although the degree of the effect of host genetics on the composition of the gut microbiome is not yet clear [16], various associations between genetics and bacterial taxa abundances have been observed. Studies have observed a consistent association between the lactase (LCT) gene, which produces the enzyme lactase that digests lactose, and the relative abundance of *Bifidobacterium*, a genus of bacteria found to produce several bioactive metabolites [17-19]. Similarly, a study on monozygotic and dizygotic twins found significant heritability of more than half of the taxa, with *Bifidobacterium* producing the greatest heritability [20]. It was concluded that minor alleles of the genetic variant *APOA5* SNP rs651821 were significantly correlated with decreased *Bifidobacterium* abundance [20].

Montgomery *et al.* [21] also revealed the impact of host genetics, specifically on gut microbiome composition prior to experimental autoimmune encephalomyelitis (EAE) onset. Their analyses revealed the considerable significance of the genotype on the taxonomic makeup of the microbiome, proposing that host genotype plays a role in gut bacteria’s effect on central nervous system autoimmunity [21]. Other studies have also revealed associations between specific SNPs and microbial abundances, including between an SNP in the ubiquitin-protein ligase E3 component n-recognin 3 (*UBR3*) gene and *Rikenellaceae*, as well as between an SNP in the spalt-like transcription factor 3 (*SALL3*) gene and *Eubacterium* [22, 23]. Tang and colleagues [24] determined 1,368 SNPs that regulate the relative abundances of 267 microbes that primarily inhabit the host gut, mucosa, and vagina.

These studies have highlighted the role of host genetics on the gut microbiome, indicating relationships within the “complex and polygenic” network of genetics that influence the taxonomic abundances of bacteria [17]. Along with impacting relative abundances of bacterial taxa, host genetics facilitate the regulation of metabolism, especially those traits related to obesity [17]. It has also been suggested that host genetics have a stronger effect on the gut microbiome composition at younger ages, with the effect decreasing over time [16]. There is a need to better understand the association between genes and gut microbes by examining the simultaneous effects of genetics and the GI environment. In addition, the effect of age on host genetics and the combined impact of genetics and the environment on the gut microbiome composition are key areas that warrant further investigations.

## 3 METABOLITE PRODUCTION BY THE GUT MICROBIOME

**Fig. (2):**
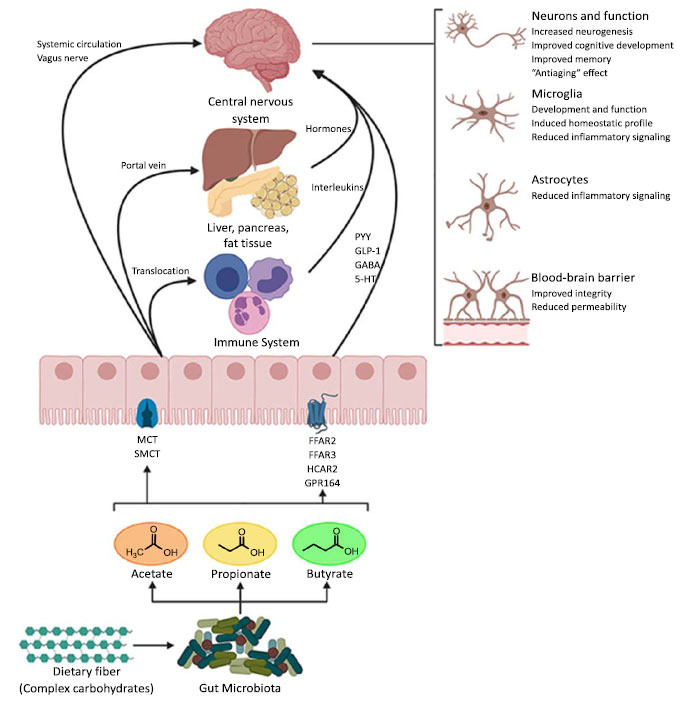
Potential pathways through which short-chain fatty acids (SCFAs) influence gut-brain communication. SCFAs are the main metabolites produced by the microbiota in the large intestine through the anaerobic fermentation of indigestible polysaccharides, such as dietary fiber and resistant starch. SCFAs might influence gut-brain communication and brain function directly or indirectly. Following their production, SCFAs are absorbed by colonocytes, mainly *via* H^+^-dependent monocarboxylate transporters (MCTs) or sodium-dependent monocarboxylate transporters (SMCTs). Through binding to G protein-coupled receptors (GPCRs), such as free fatty acid receptors 2 and 3 (FFAR2 and FFAR3), as well as GPR109a/HCAR2 (hydrocarboxylic acid receptor) and GPR164 or by inhibiting histone deacetylases, SCFAs influence intestinal mucosal immunity, and barrier integrity and function. SCFAs interaction with their receptors on enteroendocrine cells promotes indirect signaling to the brain *via* the systemic circulation or vagal pathways by inducing the secretion of gut hormones, such as glucagon-like peptide 1 (GLP1) and peptide YY (PYY), as well as GABA and serotonin (5-HT). Colon-derived SCFAs reach the systemic circulation and other tissues, leading to brown adipose tissue activation, regulation of liver mitochondrial function, increased insulin secretion by β-pancreatic cells, and whole-body energy homeostasis. Peripherally, SCFAs influence systemic inflammation mainly by inducing Treg cell differentiation and regulating the secretion of interleukins. SCFAs can cross the BBB *via* monocarboxylate transporters located on endothelial cells and influence BBB integrity by upregulating the expression of tight junction proteins. Finally, in the CNS, SCFAs also influence neuroinflammation by affecting glial cell morphology and function as well as by modulating the levels of neurotrophic factors, increasing neurogenesis, contributing to the biosynthesis of serotonin, and improving neuronal homeostasis and function. Together, the interaction of SCFAs with these gut-brain pathways can directly or indirectly affect the emotion, cognition, and pathophysiology of brain disorders. A figure of this review was created with BioRender (https://biorender.com/). Taken from Silva *et al.*, 2020, which is an open-access article distributed under the terms of the Creative Commons Attribution License (CC BY) [25]. The use, distribution or reproduction in other forums is permitted, provided the original author(s) and the copyright owner(s) are credited and that the original publication in this journal is cited in accordance with accepted academic practice.

The short-chain fatty acids (SCFAs) produced by gut microbes play a pivotal role in regulating the gut-brain axis (Fig. **2**). SCFAs are the products of colonic bacteria fermenting complex carbohydrates. Examples of SCFAs include butyric acid, acetic acid, and propionic acid. SCFAs can cross the blood-brain barrier (BBB) and influence cell physiology, as well as exert peripheral effects on microglia (Fig. **2**) [7, 25, 26]. SCFAs generally exert neuroprotective effects. However, altered production of SCFAs during gut dysbiosis can also contribute to microglia activation, aberrant alpha-synuclein deposition, and subsequent motor deficits in those who have alpha-synuclein overexpression and dysbiosis associated with PD [6, 26-28].

Research has shown that mice that had received fecal microbiota transplantation from patients with PD had increased amounts of three enzymes that are responsible for generating SCFAs, known as KEGG families (K00929 (butyrate kinase), K01034 (acetate CoA/acetoacetate CoA transferase alpha), and K01035 (acetate CoA/acetoacetate CoA transferase beta), along with an altered proportion of SCFA subtypes [7]. Specifically, the transplanted mice had higher concentrations of propionic and butyric acid and lower relative concentrations of acetic acid; alpha-synuclein overexpression in these mice may lead to this disruption in relative SCFA concentrations. These disproportionate and abnormal metabolites produced by an altered gut microbiome can cross the BBB and may have detrimental consequences for neuron cell function. The gut microbiome metabolites and microbial species that are upregulated in neurodegenerative disorders can be ideal targets for future therapies.

Excessive quantities of propionic acid can lead to stereotypy as well as hyperactivity through the disruption and alteration of neuronal phospholipid membranes [29]. The detrimental effects of propionic acid have been attributed to the lowering of monounsaturated fats, such as omega-6 fatty acids and phosphatidylethanolamine plasmalogens, while increasing saturated fats in neural membranes, thus disrupting their normal function [30]. Rats that received intracerebroventricular propionic acid injections exhibited impaired social functioning, reduced ability to complete a reversal in a T-maze, and a propensity to limit interest to a single object within a group of objects [31]. Other studies have also observed that rats treated with propionic acid had impaired spatial reversal training on the water-maze task and poor performance on the beam task, which corresponded to increased neuroinflammation in the histopathological analysis [32]. In addition, rats that received propionic acid also interacted less with each other, including being more physically distanced from one another and displaying reduced as well as impaired playful interactions [33].

PD patients, regardless of diet, background, and medication use, have increased gut concentrations of the bacterial species *Verrucomicrobiaceae* and *Akkermansia* and decreased numbers of *Prevotellaceae* and *Lachnospiraceae* [34]. This difference in gut microbiota composition might explain the differences in the proportions of different SCFAs produced by these bacteria and subsequently, the severity of central nervous system (CNS) disease.

The metabolism of certain drugs may affect treatments for neurodegenerative disease. For example, L-dopa (levodopa), the first-line medication for PD that is converted to dopamine by dopaminergic neurons to replenish low dopamine levels found in PD patients, is metabolized in the gut differently depending on the host’s gut microbial composition. Bacteria that express tyrosine decarboxylase can metabolize L-dopa to an extent, limiting its access to the brain. These tyrosine decarboxylase-positive bacteria can therefore limit the efficacy of levodopa treatments, thus requiring higher drug doses for certain subsets of PD patients [34]. Treatments that increase L-dopa bioavailability should be aimed at upregulating host tyrosine decarboxylase genes in the brain so that the drug can be converted to dopamine.

Individuals with ASD have altered gut metabolite profiles, which may play a role in the development of this neurodevelopmental disorder. 5-aminovaleric acid and taurine, both γ-aminobutyric acid type A (GABA_A_) receptor agonists, were found to be present at lower levels in the guts of mice colonized with gut microbiota from human ASD patients (Table **1**) [35]. Taurine, which can be produced by neurons and astrocytes or transported across the BBB *via* the taurine transporter, is crucial for normal brain development [36]. This finding might suggest that 5-aminovaleric acid and taurine have protective effects against the development of ASD. These results could suggest that reduced regulation from lower levels of GABA-stimulating metabolites that inhibit neural signaling may play an active role in the evolution of social developmental detriments seen in ASD. Additional studies should be carried out to determine if other GABA_A_ agonists have a similar effect. On the other hand, the glycine receptor agonist 3-aminoisobutyric acid, a degradation product of thymine, was increased in ASD mice [35]. Given that glycine and GABA receptors both inhibit neurotransmission, more research is needed to determine if an imbalance in excitatory and inhibitory neurotransmission induced by gut metabolites contributes to the development of ASD. It should be noted that glycine can also act as an agonist at excitatory NMDA glutamate receptors in some higher brain areas.

The levels of certain amino acid enzymes and their products in the guts of ASD and healthy mice differ quite significantly [35]. In the gut, Δ1-pyrroline-5-carboxylate (P5C), an intermediate of proline production, is ultimately reduced to L-proline by P5C reductase. L-proline can later be oxidized to L-glutamate by proline dehydrogenase. In healthy microbiota, L-proline typically serves as an electron acceptor to produce 5-aminovaleric acid. Research has shown that ASD mice have increased levels of proline dehydrogenase and decreased amounts of P5C reductase [35]. This imbalance results in less L-proline and consequently less 5-aminovaleric acid being produced and more L-glutamate being generated. L-glutamate acts as a neurotransmitter that links the vagus nerve to enteroendocrine cells in the intestinal lumen, allowing the gut to directly influence specific processes in the brain [37]. Given that L-glutamate stimulates neurotransmission, there is likely an association between excess excitatory neurotransmitters and the development of ASD. Therefore, gut metabolites likely play a crucial role in determining predisposition to ASD.

Another class of gut metabolites that is linked to CNS maladies are phenols, which are metabolized by the gut microbiome [38]. In the gut, the phenol tyrosine is metabolized by the microbiome to form p-cresol and subsequently sulfated by the host to form p-cresyl sulfate and 4-ethylphenyl sulfate (4EPS) [39]. 4EPS has been found to be elevated in mice with ASD as well as in schizophrenia along with children with ASD [40]. In addition, the administration of 4EPS into wild-type mice induced anxiety-like behavior [41]. p-cresyl sulfate may be a potential urinary biomarker for ASD in children and has been correlated with altered oligodendrocytes in mouse models that exhibit impaired social behaviors [42, 43]. The p-cresol has been shown to modulate dendrite development, synaptogenesis, and synapse function in hippocampal neurons *in vitro* [44]. Compared to neurotypical individuals, p-cresol has been observed in higher levels in plasma, urine, and fecal matter of ASD individuals [42, 45-49]. It has been shown that p-cresol can induce social behavior deficits that are correlated with diminished central dopamine neuron excitability in the brain’s natural reward circuit [39].

On the other hand, trimethylamine-N-oxide (TMAO), generated from choline, betaine, and carnitine by gut microbiome metabolism, has been found to have a protective effect on neural function [50]. Many other metabolites that can cause damage to neural tissue must first cross the BBB, so disruption of this barrier through processes, such as inflammation, can help propagate neurodegenerative disorders [7]. TMAO helps to strengthen the integrity of the BBB by upregulating genes that regulate the cytoskeleton, specifically actin bundle formation while downregulating genes associated with inflammation [51]. One of the BBB-associated genes upregulated by TMAO, called ANXA1, modulates and maintains the actin cytoskeleton, thus strengthening the barrier and exerting a neuroprotective effect [51-53]. TMAO is especially abundant in seafood, so a diet high in fish may be protective for patients currently living with or who are at risk for developing the neurodegenerative disease [51].

## 4 APOPTOSIS AND CELL DAMAGE

The loss of neurons in PD occurs by the accumulation of excessive alpha-synuclein. Microglia are immune cells that act to support and maintain the health of neural cells. Excess alpha-synuclein has been shown to lower microglial activation in the brain [34]. Without sufficient microglial activity, toxic metabolites and pathogens are not effectively removed from the neurons, resulting in progressive neurotoxicity and cell death, which are observed in neurodegenerative disorders, such as PD. On the other hand, excessive microglial activation may be associated with neuroinflammatory processes related to diseases like PD, which can interfere with normal T cell function.

Cytokines have long been known to play a crucial role in the inflammatory process. Helper T (Th) cell activation and cytokine release are modulated by regulatory T cells (Treg). Treg cells have been found to be less able to control the proliferation of effector T cells and cytokines, specifically the pro-inflammatory cytokines TNF-α, IL-2, IL-6, IL-10, IL-1β, and IFN-γ, in Parkinson’s disease patients [34]. This diminished regulation of pro-inflammatory factors leads to an increased level of inflammation in the body’s tissues, including neural tissue.

Th17 cells from PD patients can indirectly damage dopamine-producing brain cells by releasing IL-17, which has inflammatory cytotoxic effects. Overproduction of alpha-synuclein in the brain plays a role in upregulating a Th17 autoimmune response, causing Th17 to target dopaminergic neurons that, unlike most neurons, possess major histocompatibility complexes (MHCs) that are necessary for autoimmunity [34]. These autoimmune neurotoxic effects are indirectly facilitated by alpha-synuclein overexpression, supplementing the direct cytotoxic effects of intracellular alpha-synuclein accumulation and misfolding.

A disrupted gut microbiome may play a role in propagating autoimmune Th17 cells. Given that Th cells normally become differentiated by being exposed to microbial antigens in the thymus, it is possible that abnormal gut bacteria antigens could be interfering with this process, inducing the release of higher numbers of autoreactive Th cells into circulation that can cross the BBB to attack healthy neurons [34]. In addition, it has been observed that intestinal infection by Gram-negative bacteria can lead to mitochondrial antigen presentation in both peripheral cells and cells of the CNS, possibly inducing the activation and propagation of autoreactive cytotoxic T cells against the mitochondria of dopamine-producing neurons, ultimately leading to cell death [54].

## 5 EFFECTS ON THE CNS

Microbiome composition and the existence of different microbes influence cell differentiation and fate, including those cells involved in the CNS [55]. The gut microbiota and its metabolites are also known to regulate T cell-dependent inflammation [56-59]. Within the CNS, astrocytes are abundant in order to perform various functions, including contributing to CNS injury and disease processes [59]. These cells are not only impacted by elements within the CNS but also by factors that originate from the rest of the body as well [59]. In addition to astrocytes, microglia in the CNS are imperative for brain functioning, development, and host defense [60, 61]. The genes associated with these microglia have also been correlated with neurological disorders [61].

As previous studies have revealed connections between the GI system and the CNS, further research has continued to explore this relationship and its impact on disease processes, as well as the effects of dietary intake. Rothhammer and colleagues [59] discovered an interferon-I-aryl hydrocarbon receptor (IFN-I-AhR) axis with the capacity to limit pathogenic astrocyte functions. It was observed that IFNAR1 signaling is beneficial in astrocytes by providing anti-inflammatory properties and preventing neurodegeneration caused by astrocytes [59]. There are also implications from the AhR on the pathogenesis of immune-mediated diseases. This is caused by imbalances in “the uptake, production and/or degradation of AhR agonists,” which are produced from diet and the commensal flora [59]. These findings reveal the impact of diet and the microbiota on CNS activity and neuroinflammation.

In another study, researchers determined differences between germ-free (GF) and antibiotic-treated mice in order to reveal the impact of host microbiota on microglia function and immune response, finding that the microbiota significantly affects microglia in the CNS [61]. Complex microbiota and metabolites, such as SCFAs, were correlated with microglia homeostasis and function, with microglia deficiency partially repaired with complex microbiota. These results highlight the importance of the gut microbiota for microglia maturation and innate immune functioning, potentially providing insight into the process of diseases of the CNS [61].

Additionally, an association between a set of genes that are enhanced in hormone binding and schizophrenia has been observed [62]. Hormone binding is known to be important for the gut microbiome, as well as having associations with mental health. These findings may demonstrate a connection between microbiome diversity and schizophrenia [62]. Alterations in immunological factors and neuronal response are also potentially responsible for this relationship between the gut microbiome and schizophrenia, including the CSMD1 gene associated with schizophrenia, which is suggested to possibly impact cognitive brain functioning along with its role in inhibiting the classical complement pathway of the immune system [62].

Although the central nervous system was previously thought to be protected by the BBB, studies have revealed the possible effects of intestinal toxins in forming alpha-synuclein aggregates that travel *via* the vagus nerve to the CNS [63, 64]. Additionally, “psychobiotics” refer to a collection of probiotics that can influence the CNS and may be able to provide future benefits in the treatment of CNS disorders by targeting the gut microbiome [64-66].

## 6 THE GUT MICROBIOME’S RELATION TO AD

AD is a neurodegenerative form of dementia that leads to severe memory impairment, cognitive deterioration, and behavioral changes [67-69]. The etiology of AD has been linked with extracellular β-amyloid plaques, formation of neurofibrillary tangles, neuroinflammation, neuronal injury, and damage to neuronal synapses [70]. Declined hippocampal cognitive abilities (such as spatial learning and memory), as well as decreased long-term potentiation (LTP), have been observed in patients and animal models of AD [70-73]. However, it is important to note that at physiological levels, β-amyloid deposits have neuroprotective effects, particularly antimicrobial and antioxidant properties, that limit neuroinflammation [74].

Rezaei *et al.* [70] analyzed the effects of the gut microbiome and probiotics on AD by using β-amyloid administered rats as an animal model of the disease. Consistent with previous literature, this study revealed improved spatial performance in the rats with AD following probiotic treatment and observed an improvement in the balance of antioxidant/oxidant biomarkers and synaptic plasticity [70]. Since probiotics are known to decrease intestinal permeability, it is suspected that this contributes to the improvement in cognitive and spatial functioning following probiotic administration [70, 75]. Also, similar to the benefit of SCFAs, as previously discussed, probiotic treatment invokes an increase in SCFAs, decreased hippocampal pro-inflammatory cytokine concentration, and enhanced learning and memory as a result [70, 76]. Probiotics also work to inhibit oxidative stress by impacting the gut microbiome, thus reversing the hypothesized promotion of oxidant stressors by β-amyloid in AD [70].

## 7 ASD AND GUT HEALTH

ASDs encompass a collection of neurodevelopmental disorders which are characterized by impairments in social communication and interactions, as well as restricted and repetitive behaviors, interests, or activities [77, 78]. Due to the constantly increasing reported prevalence of ASD in children and adults and the common incidence of co-occurring GI complications in individuals diagnosed with ASD, many researchers have suggested a link between ASD and the gut microbiome as part of the gut-brain axis [39, 79-82]. In some cases, GI discomfort has been associated with autistic behaviors, and parents have noted increased behavioral problems in children with ASD following the consumption of products containing gluten and casein [83, 84]. These reported associations have motivated research demonstrating significant microbiota compositional differences between individuals with ASD *versus* neurotypical individuals. The microbial alterations in the gut were also correlated with GI distress [79, 85-90].

This relationship between gut microbiota and social behavior has been examined with multiple animal models of ASD in order to determine the disorder’s genetic etiology and possible interventional targets. Energy, proteins and amino acids, lipids and fatty acids, and redox metabolism pathways are all determined to influence ASD [91]. The gut bacteria-induced metabolic changes in ASD have been shown to impact oxidative stress, immune function, BBB permeability, and neurotransmitter production, among other effects [91]. The BTBR mouse model used by Golubeva *et al.* [80] demonstrated the significant effects of a diminished relative abundance of *Bifidobacterium* and *Blautia* species within the gut composition. The abundance variation of the BTBR gut was correlated with deficient bile formation and tryptophan (Trp) metabolism, “leaky gut,” and social deficits in these mice [80]. Ido1 and Tdo2, which are rate-limiting enzymes in the kynurenine pathway of Trp metabolism in the intestines, are downregulated in the colons of BTBR mice, thus reducing the breakdown of Trp [80]. The resulting “leaky gut,” or dysfunctional gut barrier, of these mice can lead to gut inflammation, often activating inflammation in the brain, which is a suspected contributor to ASD behaviors [80, 92]. This connection suggests a significant impact of *Bifidobacterium* and *Blautia* supply in the gut.

These bacterial species not only play a role in the transformation of bile and the expression of intestinal *Tph1*, which activates 5-HT synthesis and speeds up GI motility [80], but they also indirectly create a series of effects that impact social behaviors related to ASD. A comparison study on the gut microbial compositional profile in ASD *versus* a typically developing (TD) group similarly revealed the effects of the metabolite production in ASD symptomatology [79]. These researchers investigated various intermediate metabolites to determine variances amongst these populations. In the ASD group, phosphatidylcholine was increased, while acetaldehyde was decreased compared to the TD group. Methylselenocysteine Se-oxide and 3-(Uracil-1-yl)-L-alanine were also found in higher rates amongst the ASD group [79]. These deviations in fecal gut metabolites are suspected to relate to the pathogenesis of the gut-brain axis in ASD by altering intracellular redox and contributing to the dysfunction of the cholinergic system, suggesting a possible source of the social deficits common in those with ASD (Table **2**) [79].

The SCFAs acetate, propionate, and butyrate are the most commonly studied gut metabolites that play a role in ASD. These three SCFAs have been found in differing abundances in those with ASD compared to neurotypical groups. However, there are inconsistencies among studies regarding whether each of these is more or less abundant in ASD groups [91]. Acetate can traverse the BBB and be used as a carbon source and an appetite-signaling compound [91, 93]. Butyrate similarly serves as a carbon source and is used in the gut for energy metabolism. It can also influence epigenetic modifications that can impact processes, including apoptosis and inflammation [91, 94]. Butyrate is a known histone deacetylase (HDAC) inhibitor, and HDAC is a regulator of nuclear factor kappa B (NF-κB), a transcription factor that helps facilitate the pro-inflammatory innate immune response. Given that NF-κB is elevated in AD, PD, and ASD patients, aberrant or varying levels of butyrate in patients with gut dysbiosis may indirectly lead to dysregulation of NF-κB expression, leading to increased neuroinflammation in these patients [95]. Propionate also plays a role in various processes which modulate intestinal homeostasis and immune functions, among others (Table **2**) [91, 96, 97].

In addition to the gut microbiota, individuals with ASD have been found to exhibit “a loss of pyramidal neurons and granule cells in the hippocampus” [78], with suggested links to apoptosis. In a study on valproic acid (VPA)-treated rats as an animal model of ASD, Gao and colleagues [78] revealed increased levels of both caspase-3 and Bax, along with decreased Bcl-2 and Akt in the hippocampus of these autistic rats, all key components of the BDNF-Akt-Bcl-2 anti-apoptotic signaling pathway. By supplementing these rats with docosahexaenoic acid (DHA), these levels were reversed, thus protecting against hippocampal neuron loss [78]. The DHA supplementation also improved spatial learning and memory, two areas that are associated with a lack of social response and expression, suggesting a benefit of DHA supplementation in individuals with ASD in order to improve communication and social skills amongst this population [78].

## 8 MICROBIOME EFFECTS ON GUT PERMEABILITY

The composition of microorganisms that inhabit the human gut may have profound effects on gut permeability. Damage to the intestinal epithelial cells, which can be seen with inflammatory bowel disease (IBD), increases intestinal wall permeability and allows gut bacterial-derived toxins, such as lipopolysaccharides (LPS), to pass into the bloodstream and cause metabolic endotoxemia [98]. Transmembrane and cytoplasmic tight junction proteins, including occludin, zonula occludens (ZOs), tricellulin, cingulin, claudin, and junctional adhesion molecules (JAM), contribute to the integrity of the intestinal wall [98, 99]. ZOs, found in both epithelial and endothelial cells, are ubiquitous peripheral membrane-associated proteins essential to intestinal wall integrity. These proteins exist in various isoforms that differ based on their binding domains, including leucine zippers, SH3, and PDZ domains [98, 100,101]. This variety in binding domains allows ZOs to form scaffold networks and help connect other tight junction proteins to the cytoskeleton [98]. The transmembrane proteins occludin and claudin also perform vital roles in the maintenance of the intestinal barrier. Occludin offers tight junction structural integrity while also involving the barrier function [98, 102]. Claudin regulates the paracellular space of the intestinal wall [98, 99]. Various studies have demonstrated the effects of bacteria, SCFA, toxins, and other exposures on the regulation of ZOs, occludins, and claudins.

The probiotic *Lactobacillus rhamnosus* GG (LGG) regulated the expression of ZO-1 and occludin in order to improve epithelial barrier disruption in IBD patients [103, 104]. *Bacteroides vulgatus* and *Bacteroides dorei* have similarly yielded increased expression of ZO-1 and improved functioning of the epithelial barrier [103, 105]. Through AMPK activation, *Akkermansia muciniphila*-derived extracellular vesicles (AmEVs) have been shown to regulate occludin to improve the integrity of the intestinal barrier, with the effects of these AmEVs being eliminated after treatment with AMPK inhibitor [106]. Additionally, *Lactobacillus plantarum* treatment has led to increased expression of genes that impact ZO-1, ZO-2, occludin, and other tight junction pathway participants in order to augment the integrity of the epithelial barrier [107, 108]. These studies illustrated the benefit of bacteria and probiotic treatments on the epithelial barrier and gut permeability.

Butyrate up-regulated occludin and ZO-1 in order to both improve gut permeability and reduce inflammation [103, 109]. Stx2a toxin microinjection has also resulted in the up-regulation of ZO-2, occludin, and claudin-1, among other tight junction proteins [103, 110]. β-glucan supplementation has also been determined as beneficial for the epithelial barrier by increasing the abundance of occludin and ZO-1 and decreasing systemic endotoxemia [111]. β-glucan has also been linked to enhanced gut microbiota diversity, which can be linked to ASD and other neurological disorders, as previously discussed. Meanwhile, ovalbumin (OVA)-sensitized rats had increased microbiome diversity and presented down-regulated ZO-1, occludin, and claudin-1, -3, -5, -7, -8, -9, and -15 expression [112]. With this, the researchers concluded that OVA exposure causes intestinal barrier impairment.

As preserving the integrity of the gut barrier is imperative for maintaining homeostasis of the body [106], it is important to understand the role that tight junction proteins play in the intestinal barrier, and what types of supplements and alterations can impact gut permeability. Not only can a leaky gut and increased permeability influence the development of obesity and diabetes by allowing LPS to navigate into the bloodstream from the gut [106], but it can also create changes in the gut that impact neurological functioning.

## 9 TARGETING GUT DYSBIOSIS TO DEVELOP TREATMENT MODALITIES

The amelioration of gut dysbiosis has the potential to treat neurodegenerative disorders that may have been brought about or worsened at least partially by gut microbiome disruption. As such, there are a number of ways that gut bacterial modulation or alterations can be used to develop treatments for diseases, such as PD or AD. In addition, gut microbiota can also serve as a diagnostic tool or biomarker for several disorders. By determining correlations between specific microbial species’ abundances and disorders, gut microbiome composition can be used to diagnose various diseases [113, 114]. By serving as disease biomarkers, these microbial taxa that are known to influence the progression of disorders can serve as an interventional drug target [7].

As a therapeutic, oral administration of probiotic microbes can displace pathogenic organisms, increase gut microbiome diversity, and promote eubiosis [115]. Probiotics can also be used as a preventive therapy. In individuals predisposed to developing neurodegenerative disorders, such as a family history of CNS disorder or current diagnosis of an intestinal disorder such as ulcerative colitis, probiotics can be administered before the pathogenic effects of dysbiosis manifest in the CNS and cause damage to neurons [115]. One challenge of oral probiotic therapy is that for the beneficial bacteria to make their way to the colon alive, they must be able to survive the harsh acidic environment of the stomach. Optimizing probiotic delivery systems or genetically modifying the bacteria to withstand the gastric environment may facilitate sufficient gut colonization and subsequent eubiosis.

The effects of probiotics include immunological benefits, such as regulation of cytokine activation, activation of local macrophages, and increased production of immunoglobulin [116-118]. Non-immunological effects may include facilitated digestion, bacteriocin production, and competition with potentially pathogenic bacteria for space and resources [115]. Studies have revealed the possible benefits of probiotic treatment for disorders including PD, AD, and ASD. In a study on individuals with PD, 12-week probiotic consumption was associated with benefits on Movement Disorders Society-Unified Parkinson's Disease Rating Scale (MDS-UPDRS), high-sensitivity C-reactive protein (hs-CRP), GSH, malondialdehyde (MDA), and insulin metabolism [119]. These researchers also found potential regulation of the risk of diabetes amongst these PD patients. Similarly, a lactic acid bacteria (LAB) probiotic mixture was shown to improve neurotoxic 1-methyl-4-phenyl-1,2,3,6-tetrahydropyridine (MPTP)-impacted motor behavior through maintenance of the abundance of TH+ cells, suggesting a possible treatment modality for impaired motor behavior in individuals with PD [64]. Probiotic species that have been used effectively include enterococci, bifidobacteria, and lactobacilli. In addition, the yeast *Saccharomyces boulardii* has also been shown to be an effective probiotic. Further research should focus on how different fungal agents can be used for bolstering the health of the gut microbiome for the health of the CNS, among other benefits of a healthy gut microbiome.

Prebiotics are also efficacious at promoting a healthy gut. Prebiotics are substances that act as fuel for preexisting beneficial gut bacteria, stimulating their growth and metabolic activity [115]. Many enriched foods contain prebiotics, including fructo-oligosaccharide supplements, inulin, lactulose, and galacto-oligosaccharides [120]. An advantage that prebiotics has over probiotics is that since prebiotics are nonliving substances, there is no concern that they will be damaged by gastric, pancreatic, or bile secretions that would otherwise render them ineffective before they can be delivered to the gut, thus simplifying their delivery. Another oral treatment modality that can survive harsh GI secretions is postbiotics, defined as bioactive microbial metabolites from heat-killed microorganisms. Postbiotics exert their effect on immune cells by helping to reduce inflammation [115, 121]. By moderating the inflammatory response and augmenting the development of indigenous beneficial bacteria, postbiotic and prebiotic therapies, respectively, can help to control derangements in the gut microbiome, which can contribute to the development of neurodegenerative disease (Table **3**) [122-125].

Another method of modifying dysfunctional gut microbiomes in patients with neurodegenerative or gastrointestinal diseases is fecal microbiota transplantation (FMT). FMT has been especially efficacious in treating antibiotic-resistant *Clostridium difficile* infections, which are known to cause dysbiosis that can indirectly lead to neurotoxicity and subsequent neurodegenerative disease [7, 115]. However, further research is needed to determine how effective FMT is at curing other dysbiosis-inducing gut infections. Methods of administering FMT to patients include enema, nasogastric route, and oral capsules containing bacterial preparations [115]. Replacing a dysbiotic gut microbiome with a eubiotic one can potentially prevent neurodegenerative disease or slow down preexisting disease by eliminating the damaging and neurotoxic effects of excessive inflammatory mediator production and agents that promote alpha-synuclein misfolding that may at least partially come from gut dysbiosis. One study found that intestinal inflammation and dysbiosis were induced in healthy mice that received gut microbiota transplants from mice afflicted with ulcerative colitis [126]. However, while FMT has been advantageous in practice, there are still several adverse reactions (such as nausea and dizziness at this time) that can, at times, be deterrence and make probiotic agents a more suitable treatment method [114]. Additional studies are necessary to explore the potential therapeutic and/or preventive effects of FMTs on both chronic gastrointestinal and neurodegenerative diseases.

It has also been suggested that Western diets contribute to a lack of gut microbiome diversity, even promoting imbalance towards the pathogenic gut bacteria, which is often related to inflammatory diseases [127]. A study showed a loss of Clostridiales and Bacteroidales after following a Western diet in a mouse model [128]. By the fourth generation of these mice, who continued to follow a Western diet, the phyla Clostridiales and Bacteroidales were almost extinct. As these phyla are responsible for producing SCFAs, for those diets lacking fermentable carbohydrates, dietary SCFAs can be used as an alternative to the lacking gut microbiota as an interventional approach to autoimmune and inflammatory diseases which have anteceding gut dysbiosis [127]. For example, decreased rates of SCFAs and butyrate-producing bacteria were found in children with ASD and sleep problems due to the lack of melatonin caused by these deficits. It is, therefore, thought that treatments focusing on the gut microbiota and SCFAs may improve not only the symptoms of ASD but also co-occurring sleep disorders and problems [129].

Trp metabolism and the 5-hydroxytryptamine (5-HT), kynurenine, and AhR pathways within the gastrointestinal tract play key roles in pathophysiology and are impacted variably within a number of diseases [130]. As Trp metabolism is regulated by the gut microbiome, utilizing the bacteria that promote Trp metabolism as probiotics or aiming toward a specific pathway may serve as viable therapeutic approaches to these diseases [130]. Similarly, Wei and colleagues [55] determined that AhR facilitates the regulation of neurogenesis in the adult hippocampus. This reveals the importance of the gut-brain connection in brain aging, which is impacted by gut microbiota and metabolites. By determining the metabolic pathways that contribute to the pathology of various diseases, including those related to brain aging, these diseases can possibly be managed *via* the development of targeted interventions.

## CONCLUSION AND FUTURE DIRECTIONS

The host’s genetic background has a significant indirect impact on the development and progression of neurodegenerative disorders by altering the gut microbiome composition and relative abundances of microbial taxa. Recent studies have shown significant associations between the LCT gene and the relative abundance of *Bifidobacterium*, which is known to produce bioactive metabolites, as well as between various SNPs and specific gut microbes. Host genetics have also been shown to play a role in metabolism regulation, with possible combined effects on the environment.

Certain metabolites produced by gut bacteria can cross the BBB and cause direct damage to neurons. Alterations in the relative proportions of SCFAs, commonly found in neurodegenerative diseases, such as PD, can be harmful to neurons. In healthy patients, SCFAs contribute to microglial activation, which is neuroprotective, and reduce inflammation in organs, including the brain. The relative proportions of the different types of SCFAs differ in PD patients compared to healthy individuals. These differences correspond to the differences in gut microbiota composition found in PD patients, suggesting that higher concentrations of certain SCFAs found in PD patients, including butyric acid and propionic acid, can cause neurotoxicity and subsequent behavior changes. For example, Ossenkopp and colleagues found that rats receiving injections of propionic acid, an SCFA and enteric bacterial fermentation product, developed conditioned taste and place avoidance [131]. However, some studies have linked certain SCFAs, such as butyrate, to lower levels of neuroinflammation and improvement in the damage of motor and dopaminergic neurons found in certain neurodegenerative diseases [132]. The gut microbial metabolite trimethylamine N-oxide (TMAO) is found in higher amounts in dysbiotic intestinal systems and normally acts to augment platelet production [133-135]. This increased platelet activation in patients with gut dysbiosis is linked to broader downstream effects, such as fibrinogen and thrombin regulation, that may have detrimental effects on BBB and brain health [136]. Decreased butyrate and subsequent increase in LPS may also contribute to increased platelet activation, inflammation, and demyelination [136, 137]. Butyrate derived from gut microorganisms facilitates mitochondrial function in nerve and systemic cells by upregulating the activity of the pyruvate dehydrogenase complex, so lower levels of butyrate may contribute to lower ATP output by mitochondria and thus disrupt cell function, particularly cells in the brain that chiefly rely on pyruvate dehydrogenase complex-dependent oxidative phosphorylation for energy production [138].

Alpha-synuclein overexpression, a phenomenon that many neurodegenerative pathologies have in common, has been linked with lower levels of microglial activation. Since microglia are responsible for maintaining neuron health, the lower microglial activity would result in the build-up of toxic metabolites in neurons that can result in the destruction of neurons. On another note, SCFAs promote microglial activation, and the overproduction of certain SCFAs may be linked to microglial overactivity. PD-induced neuroinflammation can also cause the over-activation of microglia. Deranged gut microbiota can interfere with the differentiation of Th cells, which can potentially increase the frequency that T cells become autoreactive, crossing the BBB and targeting neural cells. PD patients have less effective Treg cells, which results in dysregulation of pro-inflammatory cytokine release. This pro-inflammatory state also affects brain tissue, and neuroinflammation is associated with progressive neurodegeneration.

The gut microbiome influences the differentiation of cells within the CNS, including astrocytes and microglia. Due to the connection of AhR agonists with immune-mediated diseases, it is suggested that diet and gut bacteria can influence the activity of the CNS and disease processes. Thus, future studies should focus on the effective use of probiotics as an intervention method for neurodegeneration and diseases of the CNS.

AD is often associated with decreased cognitive abilities in the hippocampus as well as decreased long-term potentiation. These two consequences of AD have shown benefits from probiotic administration in animal models, reducing increased oxidative stress. As a result, the effects of probiotics on gut permeability and β-amyloid plaques in humans require further investigation to better guide the pathogenesis and treatment of AD. Increased levels of β-amyloid are not restricted to Alzheimer’s disease but are also evident in Parkinson’s disease (especially with Lewy Bodies), surrounding motor neurons in amyotrophic lateral sclerosis, highly expressed in glioblastoma and around breast cancer cells, with BACE1 and its positive lncRNA regulator, BACE1-AS, also significantly increased in autism [139]. Elevated β-amyloid levels may be intimately linked to TLR4 signaling, including from LPS derived from a leaky gut, but also from other endogenous TLR4 ligands, such as hsp70 and HMGB1, with the initial induction of β-amyloid part of a protective response. This would suggest that factor(s) that normally act to negative feedback and limit BACE1 induction and β-amyloid may be a relevant aspect of all the disorders covered in the current review. The lack of any efficacy of anti-β-amyloid antibody treatments in AD may suggest the inappropriate conceptualization of β-amyloid in CNS disorders that warrants future investigations.

The frequent co-occurrence of GI issues in individuals with ASD has emphasized the relationship between the gut microbiome and social behaviors. Variances in gut composition and permeability can lead to inflammation in the gut and, eventually, the brain, creating impaired sociality in those with ASD. Similarly, gut metabolite differences and loss of neurons in the hippocampus in ASD can contribute to social deficits, revealing possible future treatment targets.

Tight junction proteins, such as ZOs, occludins, and claudins, are important in the maintenance of intestinal wall integrity and gut permeability. Studies have demonstrated the beneficial effects of probiotics and certain bacterial species, as well as SCFAs, such as butyrate, on the functioning of the intestinal barrier. Increased gut permeability has also been linked to changes in the gut that can impact neurological functioning.

With the potential for gut bacteria to serve as biomarkers for various diseases, certain targeted interventions have proven effective. Probiotics, prebiotics, and postbiotics, as well as fecal microbiome transplants, have been considered and utilized as treatment modalities for disorders, including those of the CNS, such as ASD. The impacts of SCFAs and Trp metabolism pathways also suggest the potential for drug and interventional targets.

Due to the significant and diverse impact of the gut microbiota and gut metabolites on the brain and processes, such as apoptosis, neuroinflammation, and oxidative stress, the management of neurodegenerative disorders, including PD, AD, and ASD, can incorporate treatments that target gut dysbiosis. By regulating the microbial composition of the gut and hence microbial metabolites, the progression of various neurological disorders may be reduced or reversed, leading to improved quality of life for affected individuals and their families.

## Figures and Tables

**Table 1:** Gut metabolites associated with ASD.

**Metabolite**	**Mechanism**	**References**
5-aminovaleric acid	• Has an inhibitory effect on neural transmission *via* GABA agonism • Present in lower amounts in the guts of ASD patients compared to healthy guts	[35]
Taurine	• GABA receptor agonist • Decreased in the guts of ASD patients • Plays a significant role in normal brain development • May delay the transition from neural excitation to inhibition that is part of normal neural development	[35]
3-aminoisobutyric acid	• Glycine receptor agonist • Abnormally increased in the guts of ASD patients • Degradation product of thymine	[35]
L-glutamate	• Produced at abnormally higher levels in the intestines of ASD patients • Excitatory neurotransmitters that may alter normal neurodevelopment and contribute to ASD development	[35] [37]
p-cresyl sulfate	• Potential biomarker of ASD • Associated with abnormal oligodendrocytes in socially impaired mice	[40]
Trimethylamine-N-oxide (TMAO)	• Neuroprotective • Upregulates genes (*e.g*., ANXA1) that strengthen the BBB	[51]

**Table 2:** Gut-derived SCFAs and their role in the gut-brain axis.

**Short-chain Fatty Acid**	**Influence on the Gut-brain Axis**	**References**
General characteristics of SCFAs	• May contribute to microglia activation, aberrant alpha-synuclein deposition, and subsequent motor deficits in patients with PD who have alpha-synuclein overexpression and dysbiosis. • Capable of crossing the BBB and influencing cell physiology. • Aid in the aggregation of alpha-synuclein, causing cytotoxicity and motor deficits that may propagate neurodegeneration. • Disproportionate relative concentrations can cross the BBB and negatively impact neuron cell function.	[7]
Phosphatidylcholine	• Increased quantity in the guts of ASD patients alters intracellular redox reactions and facilitates dysfunction of the cholinergic system.	[79]
Propionate	• Upregulated in patients with neurodegenerative disease. • Modulates intestinal homeostasis and immune function. • Alters neuron phospholipid membranes	[7, 29, 91, 96, 97]
Acetate	• Used as a carbon source. • Appetite-signaling compound. • Downregulated in the intestinal tracts of neurodegenerative disease patients.	[7, 91, 93]
Butyrate	• Used as a carbon source and for energy metabolism in the gut. • Impacts epigenetic modifications that can promote processes, such as inflammation and apoptosis. • Altered in the guts of patients with neurodegenerative disease (with varied results of increased/decreased abundance).	[7, 91, 94]

**Table 3:** Comparison of probiotics, prebiotics, and postbiotics in relation to gut metabolism.

**-**	**Description**	**Benefits/Pros**	**Disadvantages/Cons**	**References**
**Probiotics**	• “Live microorganisms that confer a health benefit when consumed in adequate amounts.” • Ex: *Enterococcus*, *Bifidobacterium*, and *Lactobacillus*	• Regulation of cytokine activation. • Activation of local macrophages. • Increased production of immunoglobin. • Facilitate digestion. • Bacteriocin production. • Competition with potentially pathogenic bacteria for space and resources. • Supplements are typically considered safe for humans.	• Higher risk of infection/ morbidity in immunocompromised patients. • Cases of bacteremia, sepsis, endocarditis, and fungal sepsis linked to certain probiotics.	[115, 123, 124]
**Prebiotics**	• “Selectively fermented dietary ingredients” that alter the gut microbiota. • Ex: fructo-oligosaccharide supplements, inulin, lactulose, and galacto-oligosaccharides, natural sources (beans, starchy fruits, soybeans, *etc*.)	• Stimulate the growth and metabolic activity of beneficial gut bacteria. • They are nonliving, so there is no possibility of being killed by gastric, pancreatic, or bile secretions, thus simplifying their delivery. • Evidence of reduced body fat content. • Potentially easy and cheap method of improving energy metabolism.	• Most research has been performed on animals with limited human data. • No reports of serious side effects in studies.	[115, 120, 124]
**Postbiotics**	• Bioactive microbial metabolites from heat-killed microorganisms. • Ex: paraprobiotics, fermented infant formula (FIF), SCFAs, peptides, enzymes, vitamins	• Facilitate the reduction of inflammation. • Can potentially manage gut barrier status caused by gut dysbiosis.	• More studies are warranted to determine the clinical effects.	[115, 122,124, 125]

## LIST OF ABBREVIATIONS

AD: Alzheimer’s Disease
ASD: Autism Spectrum Disorder
BBB: Blood-brain Barrier
CNS: Central Nervous System
EAE: Experimental Autoimmune Encephalomyelitis
FMT: Fecal Microbiota Transplantation
GI: Gastrointestinal
IBD: Inflammatory Bowel Disease
JAM: Junctional Adhesion Molecules
LTP: Long-term Potentiation
MHCs: Major Histocompatibility Complexes
PD: Parkinson’s Disease
SCFAs: Short-chain Fatty Acids
SNPs: Single Nucleotide Polymorphisms
TD: Typically Developing

## References

[1] Verhaar B.J.H., Hendriksen H.M.A., de Leeuw F.A., Doorduijn A.S., van Leeuwenstijn M., Teunissen C.E., Barkhof F., Scheltens P., Kraaij R., van Duijn C.M., Nieuwdorp M., Muller M., van der Flier W.M. (2022). Gut microbiota composition is related to AD pathology.. Front. Immunol..

[2] Konopelski P., Mogilnicka I. (2022). Biological effects of indole-3-propionic acid, a gut microbiota-derived metabolite, and its precursor tryptophan in mammals’ health and disease.. Int. J. Mol. Sci..

[3] Eshraghi R.S., Davies C., Iyengar R., Perez L., Mittal R., Eshraghi A.A. (2020). Gut-induced inflammation during development may compromise the blood-brain barrier and predispose to autism spectrum disorder.. J. Clin. Med..

[4] Eshraghi R.S., Deth R.C., Mittal R., Aranke M., Kay S.I.S., Moshiree B., Eshraghi A.A. (2018). Early disruption of the microbiome leading to decreased antioxidant capacity and epigenetic changes: Implications for the rise in autism.. Front. Cell. Neurosci..

[5] Kim C.H., Jung J., Lee Y., Kim K., Kang S., Kang G., Chu H., Kim S.Y., Lee S. (2022). Comparison of metabolites and gut microbes between patients with Parkinson’s disease and healthy individuals – a pilot clinical observational study (STROBE compliant).. Healthcare (Basel).

[6] Chen S.J., Chen C.C., Liao H.Y., Lin Y.T., Wu Y.W., Liou J.M., Wu M.S., Kuo C.H., Lin C.H. (2022). Association of fecal and plasma levels of short-chain fatty acids with gut microbiota and clinical severity in patients with Parkinson disease.. Neurology.

[7] Sampson T.R., Debelius J.W., Thron T., Janssen S., Shastri G.G., Ilhan Z.E., Challis C., Schretter C.E., Rocha S., Gradinaru V., Chesselet M.F., Keshavarzian A., Shannon K.M., Krajmalnik-Brown R., Wittung-Stafshede P., Knight R., Mazmanian S.K. (2016). Gut microbiota regulate motor deficits and neuroinflammation in a model of Parkinson’s disease.. Cell.

[8] Brody H. (2020). The gut microbiome.. Nature.

[9] Cresci G.A., Bawden E. (2015). Gut microbiome.. Nutr. Clin. Pract..

[10] Lozupone C.A., Stombaugh J.I., Gordon J.I., Jansson J.K., Knight R. (2012). Diversity, stability and resilience of the human gut microbiota.. Nature.

[11] Shreiner A.B., Kao J.Y., Young V.B. (2015). The gut microbiome in health and in disease.. Curr. Opin. Gastroenterol..

[12] Human Microbiome Project Consortium (2012). Structure, function and diversity of the healthy human microbiome.. Nature.

[13] Manor O., Dai C.L., Kornilov S.A., Smith B., Price N.D., Lovejoy J.C., Gibbons S.M., Magis A.T. (2020). Health and disease markers correlate with gut microbiome composition across thousands of people.. Nat. Commun..

[14] Wu Y.T., Shen S.J., Liao K.F., Huang C.Y. (2022). Dietary plant and animal protein sources oppositely modulate fecal Bilophila and Lachnoclostridium in vegetarians and omnivores.. Microbiol. Spectr..

[15] Tanes C., Bittinger K., Gao Y., Friedman E.S., Nessel L., Paladhi U.R., Chau L., Panfen E., Fischbach M.A., Braun J., Xavier R.J., Clish C.B., Li H., Bushman F.D., Lewis J.D., Wu G.D. (2021). Role of dietary fiber in the recovery of the human gut microbiome and its metabolome.. Cell Host Microbe.

[16] Cahana I., Iraqi F.A. (2020). Impact of host genetics on gut microbiome: Take‐home lessons from human and mouse studies.. Animal Model. Exp. Med..

[17] Kurilshikov A., Medina-Gomez C., Bacigalupe R., Radjabzadeh D., Wang J., Demirkan A., Le Roy C.I., Raygoza Garay J.A., Finnicum C.T., Liu X., Zhernakova D.V., Bonder M.J., Hansen T.H., Frost F., Rühlemann M.C., Turpin W., Moon J.Y., Kim H.N., Lüll K., Barkan E., Shah S.A., Fornage M., Szopinska-Tokov J., Wallen Z.D., Borisevich D., Agreus L., Andreasson A., Bang C., Bedrani L., Bell J.T., Bisgaard H., Boehnke M., Boomsma D.I., Burk R.D., Claringbould A., Croitoru K., Davies G.E., van Duijn C.M., Duijts L., Falony G., Fu J., van der Graaf A., Hansen T., Homuth G., Hughes D.A., Ijzerman R.G., Jackson M.A., Jaddoe V.W.V., Joossens M., Jørgensen T., Keszthelyi D., Knight R., Laakso M., Laudes M., Launer L.J., Lieb W., Lusis A.J., Masclee A.A.M., Moll H.A., Mujagic Z., Qibin Q., Rothschild D., Shin H., Sørensen S.J., Steves C.J., Thorsen J., Timpson N.J., Tito R.Y., Vieira-Silva S., Völker U., Völzke H., Võsa U., Wade K.H., Walter S., Watanabe K., Weiss S., Weiss F.U., Weissbrod O., Westra H.J., Willemsen G., Payami H., Jonkers D.M.A.E., Arias Vasquez A., de Geus E.J.C., Meyer K.A., Stokholm J., Segal E., Org E., Wijmenga C., Kim H.L., Kaplan R.C., Spector T.D., Uitterlinden A.G., Rivadeneira F., Franke A., Lerch M.M., Franke L., Sanna S., D’Amato M., Pedersen O., Paterson A.D., Kraaij R., Raes J., Zhernakova A. (2021). Large-scale association analyses identify host factors influencing human gut microbiome composition.. Nat. Genet..

[18] Schmidt V., Enav H., Spector T.D., Youngblut N.D., Ley R.E. (2020). Strain-level analysis of Bifidobacterium spp. from gut microbiomes of adults with differing lactase persistence genotypes.. mSystems.

[19] Kolde R., Franzosa E.A., Rahnavard G., Hall A.B., Vlamakis H., Stevens C., Daly M.J., Xavier R.J., Huttenhower C. (2018). Host genetic variation and its microbiome interactions within the Human Microbiome Project.. Genome Med..

[20] Lim M.Y., You H.J., Yoon H.S., Kwon B., Lee J.Y., Lee S., Song Y.M., Lee K., Sung J., Ko G. (2017). The effect of heritability and host genetics on the gut microbiota and metabolic syndrome.. Gut.

[21] Montgomery T.L., Künstner A., Kennedy J.J., Fang Q., Asarian L., Culp-Hill R., D’Alessandro A., Teuscher C., Busch H., Krementsov D.N. (2020). Interactions between host genetics and gut microbiota determine susceptibility to CNS autoimmunity.. Proc. Natl. Acad. Sci. USA.

[22] Turpin W., Espin-Garcia O., Xu W., Silverberg M.S., Kevans D., Smith M.I., Guttman D.S., Griffiths A., Panaccione R., Otley A., Xu L., Shestopaloff K., Moreno-Hagelsieb G., Paterson A.D., Croitoru K. (2016). Association of host genome with intestinal microbial composition in a large healthy cohort.. Nat. Genet..

[23] Bubier J.A., Chesler E.J., Weinstock G.M. (2021). Host genetic control of gut microbiome composition.. Mamm. Genome.

[24] Tang J., Wu X., Mou M., Wang C., Wang L., Li F., Guo M., Yin J., Xie W., Wang X., Wang Y., Ding Y., Xue W., Zhu F. (2021). GIMICA: Host genetic and immune factors shaping human microbiota.. Nucleic Acids Res..

[25] Silva Y.P., Bernardi A., Frozza R.L. (2020). The role of short-chain fatty acids from gut microbiota in gut-brain communication.. Front. Endocrinol. (Lausanne).

[26] Mitchell R.W., On N.H., Del Bigio M.R., Miller D.W., Hatch G.M. (2011). Fatty acid transport protein expression in human brain and potential role in fatty acid transport across human brain microvessel endothelial cells.. J. Neurochem..

[27] Lee J., Venna V.R., Durgan D.J., Shi H., Hudobenko J., Putluri N., Petrosino J., McCullough L.D., Bryan R.M. (2020). Young versus aged microbiota transplants to germ-free mice: Increased short-chain fatty acids and improved cognitive performance.. Gut Microbes.

[28] Unger M.M., Spiegel J., Dillmann K.U., Grundmann D., Philippeit H., Bürmann J., Faßbender K., Schwiertz A., Schäfer K.H. (2016). Short chain fatty acids and gut microbiota differ between patients with Parkinson’s disease and age-matched controls.. Parkinsonism Relat. Disord..

[29] Thomas R.H., Meeking M.M., Mepham J.R., Tichenoff L., Possmayer F., Liu S., MacFabe D.F. (2012). The enteric bacterial metabolite propionic acid alters brain and plasma phospholipid molecular species: Further development of a rodent model of autism spectrum disorders.. J. Neuroinflammation.

[30] Thomas R.H., Foley K.A., Mepham J.R., Tichenoff L.J., Possmayer F., MacFabe D.F. (2010). Altered brain phospholipid and acylcarnitine profiles in propionic acid infused rodents: Further development of a potential model of autism spectrum disorders.. J. Neurochem..

[31] MacFabe D.F., Cain N.E., Boon F., Ossenkopp K.P., Cain D.P. (2011). Effects of the enteric bacterial metabolic product propionic acid on object-directed behavior, social behavior, cognition, and neuroinflammation in adolescent rats: Relevance to autism spectrum disorder.. Behav. Brain Res..

[32] Shultz S.R., MacFabe D.F., Martin S., Jackson J., Taylor R., Boon F., Ossenkopp K.P., Cain D.P. (2009). Intracerebroventricular injections of the enteric bacterial metabolic product propionic acid impair cognition and sensorimotor ability in the Long–Evans rat: Further development of a rodent model of autism.. Behav. Brain Res..

[33] Shultz S.R., MacFabe D.F., Ossenkopp K.P., Scratch S., Whelan J., Taylor R., Cain D.P. (2008). Intracerebroventricular injection of propionic acid, an enteric bacterial metabolic end-product, impairs social behavior in the rat: Implications for an animal model of autism.. Neuropharmacology.

[34] Zheng W., He R., Yan Z., Huang Y., Huang W., Cai Z., Su Y., Liu S., Deng Y., Wang Q., Xie H. (2020). Regulation of immune-driven pathogenesis in Parkinson’s disease by gut microbiota.. Brain Behav. Immun..

[35] Sharon G., Cruz N.J., Kang D.W., Gandal M.J., Wang B., Kim Y.M., Zink E.M., Casey C.P., Taylor B.C., Lane C.J., Bramer L.M., Isern N.G., Hoyt D.W., Noecker C., Sweredoski M.J., Moradian A., Borenstein E., Jansson J.K., Knight R., Metz T.O., Lois C., Geschwind D.H., Krajmalnik-Brown R., Mazmanian S.K., Mazmanian S.K. (2019). Human gut microbiota from autism spectrum disorder promote behavioral symptoms in mice.. Cell.

[36] Mersman B., Zaidi W., Syed N.I., Xu F. (2020). Taurine promotes neurite outgrowth and synapse development of both vertebrate and invertebrate central neurons.. Front. Synaptic Neurosci..

[37] Kaelberer M.M., Buchanan K.L., Klein M.E., Barth B.B., Montoya M.M., Shen X., Bohórquez D.V. (2018). A gut-brain neural circuit for nutrient sensory transduction.. Science.

[38] Needham B.D., Kaddurah-Daouk R., Mazmanian S.K. (2020). Gut microbial molecules in behavioural and neurodegenerative conditions.. Nat. Rev. Neurosci..

[39] Bermudez-Martin P., Becker J.A.J., Caramello N., Fernandez S.P., Costa-Campos R., Canaguier J., Barbosa S., Martinez-Gili L., Myridakis A., Dumas M.E., Bruneau A., Cherbuy C., Langella P., Callebert J., Launay J.M., Chabry J., Barik J., Le Merrer J., Glaichenhaus N., Davidovic L. (2021). The microbial metabolite p-Cresol induces autistic-like behaviors in mice by remodeling the gut microbiota.. Microbiome.

[40] Needham B.D., Adame M.D., Serena G., Rose D.R., Preston G.M., Conrad M.C., Campbell A.S., Donabedian D.H., Fasano A., Ashwood P., Mazmanian S.K. (2021). Plasma and fecal metabolite profiles in autism spectrum disorder.. Biol. Psychiatry.

[41] Hsiao E.Y., McBride S.W., Hsien S., Sharon G., Hyde E.R., McCue T., Codelli J.A., Chow J., Reisman S.E., Petrosino J.F., Patterson P.H., Mazmanian S.K. (2013). Microbiota modulate behavioral and physiological abnormalities associated with neurodevelopmental disorders.. Cell.

[42] Gabriele S., Sacco R., Cerullo S., Neri C., Urbani A., Tripi G., Malvy J., Barthelemy C., Bonnet-Brihault F., Persico A.M. (2014). Urinary p -cresol is elevated in young French children with autism spectrum disorder: A replication study.. Biomarkers.

[43] Gacias M., Gaspari S., Santos P.M.G., Tamburini S., Andrade M., Zhang F., Shen N., Tolstikov V., Kiebish M.A., Dupree J.L., Zachariou V., Clemente J.C., Casaccia P. (2016). Microbiota-driven transcriptional changes in prefrontal cortex override genetic differences in social behavior.. eLife.

[44] Guzmán-Salas S., Weber A., Malci A., Lin X., Herrera-Molina R., Cerpa W., Dorador C., Signorelli J., Zamorano P. (2022). The metaboliteP ‐cresol impairs dendritic development, synaptogenesis, and synapse function in hippocampal neurons: Implications for autism spectrum disorder.. J. Neurochem..

[45] Daneberga Z., Nakazawa-Miklasevica M., Berga-Svitina E., Murmane D., Isarova D., Cupane L., Masinska M., Nartisa I., Lazdane A., Miklasevics E. (2021). Urinary organic acids spectra in children with altered gut microbiota composition and autistic spectrum disorder.. Nord. J. Psychiatry.

[46] Kang D.W., Adams J.B., Vargason T., Santiago M., Hahn J., Krajmalnik-Brown R. (2020). Distinct fecal and plasma metabolites in children with autism spectrum disorders and their modulation after microbiota transfer therapy.. MSphere.

[47] Gevi F., Belardo A., Zolla L. (2020). A metabolomics approach to investigate urine levels of neurotransmitters and related metabolites in autistic children.. Biochim. Biophys. Acta Mol. Basis Dis..

[48] Kang D.W., Ilhan Z.E., Isern N.G., Hoyt D.W., Howsmon D.P., Shaffer M., Lozupone C.A., Hahn J., Adams J.B., Krajmalnik-Brown R. (2018). Differences in fecal microbial metabolites and microbiota of children with autism spectrum disorders.. Anaerobe.

[49] Altieri L., Neri C., Sacco R., Curatolo P., Benvenuto A., Muratori F., Santocchi E., Bravaccio C., Lenti C., Saccani M., Rigardetto R., Gandione M., Urbani A., Persico A.M. (2011). Urinary p -cresol is elevated in small children with severe autism spectrum disorder.. Biomarkers.

[50] Velasquez M., Ramezani A., Manal A., Raj D. (2016). Trimethylamine N-oxide: The good, the bad and the unknown.. Toxins (Basel).

[51] Hoyles L., Pontifex M.G., Rodriguez-Ramiro I., Anis-Alavi M.A., Jelane K.S., Snelling T., Solito E., Fonseca S., Carvalho A.L., Carding S.R., Müller M., Glen R.C., Vauzour D., McArthur S. (2021). Regulation of blood–brain barrier integrity by microbiome-associated methylamines and cognition by trimethylamine N-oxide.. Microbiome.

[52] Gobbetti T., Cooray S.N. (2016). Annexin A1 and resolution of inflammation: Tissue repairing properties and signalling signature.. Biol. Chem..

[53] Cristante E., McArthur S., Mauro C., Maggioli E., Romero I.A., Wylezinska-Arridge M., Couraud P.O., Lopez-Tremoleda J., Christian H.C., Weksler B.B., Malaspina A., Solito E. (2013). Identification of an essential endogenous regulator of blood–brain barrier integrity, and its pathological and therapeutic implications.. Proc. Natl. Acad. Sci. USA.

[54] Matheoud D., Cannon T., Voisin A., Penttinen A.M., Ramet L., Fahmy A.M., Ducrot C., Laplante A., Bourque M.J., Zhu L., Cayrol R., Le Campion A., McBride H.M., Gruenheid S., Trudeau L.E., Desjardins M. (2019). Intestinal infection triggers Parkinson’s disease-like symptoms in Pink1−/− mice.. Nature.

[55] Wei G.Z., Martin K.A., Xing P.Y., Agrawal R., Whiley L., Wood T.K., Hejndorf S., Ng Y.Z., Low J.Z.Y., Rossant J., Nechanitzky R., Holmes E., Nicholson J.K., Tan E.K., Matthews P.M., Pettersson S. (2021). Tryptophan-metabolizing gut microbes regulate adult neurogenesis via the aryl hydrocarbon receptor.. Proc. Natl. Acad. Sci. USA.

[56] Agirman G., Yu K.B., Hsiao E.Y. (2021). Signaling inflammation across the gut-brain axis.. Science.

[57] Campos-Acuña J., Elgueta D., Pacheco R. (2019). T-cell-driven inflammation as a mediator of the gut-brain axis involved in Parkinson’s disease.. Front. Immunol..

[58] Singh V., Roth S., Llovera G., Sadler R., Garzetti D., Stecher B., Dichgans M., Liesz A. (2016). Microbiota dysbiosis controls the neuroinflammatory response after stroke.. J. Neurosci..

[59] Rothhammer V., Mascanfroni I.D., Bunse L., Takenaka M.C., Kenison J.E., Mayo L., Chao C.C., Patel B., Yan R., Blain M., Alvarez J.I., Kébir H., Anandasabapathy N., Izquierdo G., Jung S., Obholzer N., Pochet N., Clish C.B., Prinz M., Prat A., Antel J., Quintana F.J. (2016). Type I interferons and microbial metabolites of tryptophan modulate astrocyte activity and central nervous system inflammation via the aryl hydrocarbon receptor.. Nat. Med..

[60] Wolf S.A., Boddeke H.W.G.M., Kettenmann H. (2017). Microglia in physiology and disease.. Annu. Rev. Physiol..

[61] Erny D., Hrabě de Angelis A.L., Jaitin D., Wieghofer P., Staszewski O., David E., Keren-Shaul H., Mahlakoiv T., Jakobshagen K., Buch T., Schwierzeck V., Utermöhlen O., Chun E., Garrett W.S., McCoy K.D., Diefenbach A., Staeheli P., Stecher B., Amit I., Prinz M. (2015). Host microbiota constantly control maturation and function of microglia in the CNS.. Nat. Neurosci..

[62] Martins-Silva T., Salatino-Oliveira A., Genro J.P., Meyer F.D.T., Li Y., Rohde L.A., Hutz M.H., Tovo-Rodrigues L. (2021). Host genetics influences the relationship between the gut microbiome and psychiatric disorders.. Prog. Neuropsychopharmacol. Biol. Psychiatry.

[63] Santos S.F., de Oliveira H.L., Yamada E.S., Neves B.C., Pereira A. (2019). The gut and Parkinson’s disease—a bidirectional pathway.. Front. Neurol..

[64] Perez Visñuk D., Savoy de Giori G., LeBlanc J.G., de Moreno de LeBlanc A. (2020). Neuroprotective effects associated with immune modulation by selected lactic acid bacteria in a Parkinson’s disease model.. Nutrition.

[65] Cheng L.H., Liu Y.W., Wu C.C., Wang S., Tsai Y.C. (2019). Psychobiotics in mental health, neurodegenerative and neurodevelopmental disorders.. Yao Wu Shi Pin Fen Xi.

[66] Cerdó T., Ruíz A., Suárez A., Campoy C. (2017). Probiotic, prebiotic, and brain development.. Nutrients.

[67] Tahami Monfared A.A., Byrnes M.J., White L.A., Zhang Q. (2022). Alzheimer’s disease: Epidemiology and clinical progression.. Neurol. Ther..

[68] Fisher R.A., Miners J.S., Love S. (2022). Pathological changes within the cerebral vasculature in Alzheimer’s disease: New perspectives.. Brain Pathol..

[69] Scheltens P., De Strooper B., Kivipelto M., Holstege H., Chételat G., Teunissen C.E., Cummings J., van der Flier W.M. (2021). Alzheimer’s disease.. Lancet.

[70] Rezaei A.Z., Sepehri G., Salami M. (2019). Probiotic treatment improves the impaired spatial cognitive performance and restores synaptic plasticity in an animal model of Alzheimer’s disease.. Behav. Brain Res..

[71] Babür E., Tan B., Delibaş S., Yousef M., Dursun N., Süer C. (2019). Depotentiation of long-term potentiation is associated with epitope-specific tau hyper-/hypophosphorylation in the hippocampus of adult rats.. J. Mol. Neurosci..

[72] Athari N.A.S., Djazayeri A., Safa M., Azami K., Djalali M., Sharifzadeh M., Vafa M. (2017). Probiotics improve insulin resistance status in an experimental model of Alzheimer’s disease.. Med. J. Islam. Repub. Iran.

[73] Yamin G. (2009). NMDA receptor-dependent signaling pathways that underlie amyloid β-protein disruption of LTP in the hippocampus.. J. Neurosci. Res..

[74] Wiatrak B., Jawień P., Matuszewska A., Szeląg A., Kubis-Kubiak A. (2022). Effect of amyloid-β on the redox system activity in SH-SY5Y cells preincubated with lipopolysaccharide or co-cultured with microglia cells.. Biomed. Pharmacother..

[75] Hemert S.V., Ormel G. (2014). Influence of the multispecies probiotic Ecologic® BARRIER on parameters of intestinal barrier function.. Food Nutr. Sci..

[76] Romo-Araiza A., Gutiérrez-Salmeán G., Galván E.J., Hernández-Frausto M., Herrera-López G., Romo-Parra H., García-Contreras V., Fernández-Presas A.M., Jasso-Chávez R., Borlongan C.V., Ibarra A. (2018). Probiotics and prebiotics as a therapeutic strategy to improve memory in a model of middle-aged rats.. Front. Aging Neurosci..

[77] American Psychiatric Association (2013). Diagnostic and Statistical Manual of Mental Disorders..

[78] Gao J., Wang X., Sun H., Cao Y., Liang S., Wang H., Wang Y., Yang F., Zhang F., Wu L. (2016). Neuroprotective effects of docosahexaenoic acid on hippocampal cell death and learning and memory impairments in a valproic acid‐induced rat autism model.. Int. J. Dev. Neurosci..

[79] Dan Z., Mao X., Liu Q., Guo M., Zhuang Y., Liu Z., Chen K., Chen J., Xu R., Tang J., Qin L., Gu B., Liu K., Su C., Zhang F., Xia Y., Hu Z., Liu X. (2020). Altered gut microbial profile is associated with abnormal metabolism activity of Autism Spectrum Disorder.. Gut Microbes.

[80] Golubeva A.V., Joyce S.A., Moloney G., Burokas A., Sherwin E., Arboleya S., Flynn I., Khochanskiy D., Moya-Pérez A., Peterson V., Rea K., Murphy K., Makarova O., Buravkov S., Hyland N.P., Stanton C., Clarke G., Gahan C.G.M., Dinan T.G., Cryan J.F. (2017). Microbiota-related changes in bile acid & tryptophan metabolism are associated with gastrointestinal dysfunction in a mouse model of autism.. EBioMedicine.

[81] Liu Z., Mao X., Dan Z., Pei Y., Xu R., Guo M., Liu K., Zhang F., Chen J., Su C., Zhuang Y., Tang J., Xia Y., Qin L., Hu Z., Liu X. (2021). Gene variations in Autism Spectrum Disorder are associated with alternation of gut microbiota, metabolites and cytokines.. Gut Microbes.

[82] Sabit H., Tombuloglu H., Rehman S., Almandil N.B., Cevik E., Abdel-Ghany S., Rashwan S., Abasiyanik M.F., Yee Waye M.M. (2021). Gut microbiota metabolites in autistic children: An epigenetic perspective.. Heliyon.

[83] Jyonouchi H., Sun S., Itokazu N. (2002). Innate immunity associated with inflammatory responses and cytokine production against common dietary proteins in patients with autism spectrum disorder.. Neuropsychobiology.

[84] MacFabe D., Cain D., Rodriguezcapote K., Franklin A., Hoffman J., Boon F., Taylor A., Kavaliers M., Ossenkopp K. (2007). Neurobiological effects of intraventricular propionic acid in rats: Possible role of short chain fatty acids on the pathogenesis and characteristics of autism spectrum disorders.. Behav. Brain Res..

[85] De Angelis M., Piccolo M., Vannini L., Siragusa S., De Giacomo A., Serrazzanetti D.I., Cristofori F., Guerzoni M.E., Gobbetti M., Francavilla R. (2013). Fecal microbiota and metabolome of children with autism and pervasive developmental disorder not otherwise specified.. PLoS One.

[86] D’Eufemia P., Celli M., Finocchiaro R., Pacifico L., Viozzi L., Zaccagnini M., Cardi E., Giardini O. (1996). Abnormal intestinal permeability in children with autism.. Acta Paediatr..

[87] Kang D.W., Park J.G., Ilhan Z.E., Wallstrom G., LaBaer J., Adams J.B., Krajmalnik-Brown R. (2013). Reduced incidence of Prevotella and other fermenters in intestinal microflora of autistic children.. PLoS One.

[88] Luna R.A., Oezguen N., Balderas M., Venkatachalam A., Runge J.K., Versalovic J., Veenstra-VanderWeele J., Anderson G.M., Savidge T., Williams K.C. (2017). Distinct microbiome-neuroimmune signatures correlate with functional abdominal pain in children with autism spectrum disorder.. Cell. Mol. Gastroenterol. Hepatol..

[89] McElhanon B.O., McCracken C., Karpen S., Sharp W.G. (2014). Gastrointestinal symptoms in autism spectrum disorder: A meta-analysis.. Pediatrics.

[90] Tomova A., Husarova V., Lakatosova S., Bakos J., Vlkova B., Babinska K., Ostatnikova D. (2015). Gastrointestinal microbiota in children with autism in Slovakia.. Physiol. Behav..

[91] Peralta-Marzal L.N., Prince N., Bajic D., Roussin L., Naudon L., Rabot S., Garssen J., Kraneveld A.D., Perez-Pardo P. (2021). The impact of gut microbiota-derived metabolites in autism spectrum disorders.. Int. J. Mol. Sci..

[92] Kociszewska D., Vlajkovic S.M. (2022). The association of inflammatory gut diseases with neuroinflammatory and auditory disorders.. Front. Biosci. (Elite Ed.).

[93] Frost G., Sleeth M.L., Sahuri-Arisoylu M., Lizarbe B., Cerdan S., Brody L., Anastasovska J., Ghourab S., Hankir M., Zhang S., Carling D., Swann J.R., Gibson G., Viardot A., Morrison D., Louise T.E., Bell J.D. (2014). The short-chain fatty acid acetate reduces appetite via a central homeostatic mechanism.. Nat. Commun..

[94] Morimoto M., Hashimoto T., Tsuda Y., Nakatsu T., Kitaoka T., Kyotani S. (2020). Assessment of oxidative stress in autism spectrum disorder using reactive oxygen metabolites and biological antioxidant potential.. PLoS One.

[95] Greene W.C., Chen L.F. (2004). Regulation of NF-kappaB action by reversible acetylation.. Novartis Found. Symp..

[96] Nankova B.B., Agarwal R., MacFabe D.F., La Gamma E.F. (2014). Enteric bacterial metabolites propionic and butyric acid modulate gene expression, including CREB-dependent catecholaminergic neurotransmission, in PC12 cells-possible relevance to autism spectrum disorders.. PLoS One.

[97] Al-Lahham S.H., Peppelenbosch M.P., Roelofsen H., Vonk R.J., Venema K. (2010). Biological effects of propionic acid in humans; metabolism, potential applications and underlying mechanisms.. Biochim. Biophys. Acta Mol. Cell Biol. Lipids.

[98] Chelakkot C., Ghim J., Ryu S.H. (2018). Mechanisms regulating intestinal barrier integrity and its pathological implications.. Exp. Mol. Med..

[99] Günzel D., Yu A.S.L. (2013). Claudins and the modulation of tight junction permeability.. Physiol. Rev..

[100] Beatch M., Jesaitis L.A., Gallin W.J., Goodenough D.A., Stevenson B.R. (1996). The tight junction protein ZO-2 contains three PDZ (PSD-95/Discs-Large/ZO-1) domains and an alternatively spliced region.. J. Biol. Chem..

[101] Itoh M., Furuse M., Morita K., Kubota K., Saitou M., Tsukita S. (1999). Direct binding of three tight junction-associated MAGUKs, ZO-1, ZO-2, and ZO-3, with the COOH termini of claudins.. J. Cell Biol..

[102] Feldman G., Mullin J., Ryan M. (2005). Occludin: Structure, function and regulation.. Adv. Drug Deliv. Rev..

[103] Allam-Ndoul B., Castonguay-Paradis S., Veilleux A. (2020). Gut microbiota and intestinal trans-epithelial permeability.. Int. J. Mol. Sci..

[104] Han X., Lee A., Huang S., Gao J., Spence J.R., Owyang C. (2019). Lactobacillus rhamnosus GG prevents epithelial barrier dysfunction induced by interferon-gamma and fecal supernatants from irritable bowel syndrome patients in human intestinal enteroids and colonoids.. Gut Microbes.

[105] Yoshida N., Emoto T., Yamashita T., Watanabe H., Hayashi T., Tabata T., Hoshi N., Hatano N., Ozawa G., Sasaki N., Mizoguchi T., Amin H.Z., Hirota Y., Ogawa W., Yamada T., Hirata K. (2018). Bacteroides vulgatus and Bacteroides dorei reduce gut microbial lipopolysaccharide production and inhibit atherosclerosis.. Circulation.

[106] Chelakkot C., Choi Y., Kim D.K., Park H.T., Ghim J., Kwon Y., Jeon J., Kim M.S., Jee Y.K., Gho Y.S., Park H.S., Kim Y.K., Ryu S.H. (2018). Akkermansia muciniphila-derived extracellular vesicles influence gut permeability through the regulation of tight junctions.. Exp. Mol. Med..

[107] Anderson R.C., Cookson A.L., McNabb W.C., Park Z., McCann M.J., Kelly W.J., Roy N.C. (2010). Lactobacillus plantarum MB452 enhances the function of the intestinal barrier by increasing the expression levels of genes involved in tight junction formation.. BMC Microbiol..

[108] Bhattarai Y. (2018). Microbiota-gut-brain axis: Interaction of gut microbes and their metabolites with host epithelial barriers.. Neurogastroenterol. Motil..

[109] Ma X., Fan P.X., Li L.S., Qiao S.Y., Zhang G.L., Li D.F. (2012). Butyrate promotes the recovering of intestinal wound healing through its positive effect on the tight junctions.. J. Anim. Sci..

[110] Pradhan S., Karve S.S., Weiss A.A., Hawkins J., Poling H.M., Helmrath M.A., Wells J.M., McCauley H.A. (2020). Tissue responses to Shiga toxin in human intestinal organoids.. Cell. Mol. Gastroenterol. Hepatol..

[111] Shi H., Yu Y., Lin D., Zheng P., Zhang P., Hu M., Wang Q., Pan W., Yang X., Hu T., Li Q., Tang R., Zhou F., Zheng K., Huang X.F. (2020). β-glucan attenuates cognitive impairment via the gut-brain axis in diet-induced obese mice.. Microbiome.

[112] Tulyeu J., Kumagai H., Jimbo E., Watanabe S., Yokoyama K., Cui L., Osaka H., Mieno M., Yamagata T. (2019). Probiotics prevents sensitization to oral antigen and subsequent increases in intestinal tight junction permeability in juvenile-young adult rats.. Microorganisms.

[113] Davenport E.R., Sanders J.G., Song S.J., Amato K.R., Clark A.G., Knight R. (2017). The human microbiome in evolution.. BMC Biol..

[114] Wang X., Zhang A., Miao J., Sun H., Yan G., Wu F., Wang X. (2018). Gut microbiota as important modulator of metabolism in health and disease.. RSC Advances.

[115] Gagliardi A., Totino V., Cacciotti F., Iebba V., Neroni B., Bonfiglio G., Trancassini M., Passariello C., Pantanella F., Schippa S. (2018). Rebuilding the gut microbiota ecosystem.. Int. J. Environ. Res. Public Health.

[116] Manzoor S., Wani S.M., Ahmad Mir S., Rizwan D. (2022). Role of probiotics and prebiotics in mitigation of different diseases.. Nutrition.

[117] Chen M., Liu C., Dai M., Wang Q., Li C., Hung W. (2022). Bifidobacterium lactis BL-99 modulates intestinal inflammation and functions in zebrafish models.. PLoS One.

[118] Lu J., Lu L., Yu Y., Baranowski J., Claud E.C. (2020). Maternal administration of probiotics promotes brain development and protects offspring’s brain from postnatal inflammatory insults in C57/BL6J mice.. Sci. Rep..

[119] Tamtaji O.R., Taghizadeh M., Daneshvar Kakhaki R., Kouchaki E., Bahmani F., Borzabadi S., Oryan S., Mafi A., Asemi Z. (2019). Clinical and metabolic response to probiotic administration in people with Parkinson’s disease: A randomized, double-blind, placebo-controlled trial.. Clin. Nutr..

[120] Chudzik A., Orzyłowska A., Rola R., Stanisz G.J. (2021). Probiotics, prebiotics and postbiotics on mitigation of depression symptoms: Modulation of the brain-gut-microbiome axis.. Biomolecules.

[121] Żółkiewicz J., Marzec A., Ruszczyński M., Feleszko W. (2020). Postbiotics— a step beyond pre- and probiotics.. Nutrients.

[122] Gu Z., Meng S., Wang Y., Lyu B., Li P., Shang N. (2022). A novel bioactive postbiotics: From microbiota-derived extracellular nanoparticles to health promoting.. Crit. Rev. Food Sci. Nutr..

[123] Hill C., Guarner F., Reid G., Gibson G.R., Merenstein D.J., Pot B., Morelli L., Canani R.B., Flint H.J., Salminen S., Calder P.C., Sanders M.E. (2014). The international scientific association for probiotics and prebiotics consensus statement on the scope and appropriate use of the term probiotic.. Nat. Rev. Gastroenterol. Hepatol..

[124] Vallianou N., Stratigou T., Christodoulatos G.S., Tsigalou C., Dalamaga M. (2020). Probiotics, prebiotics, synbiotics, postbiotics, and obesity: Current evidence, controversies, and perspectives.. Curr. Obes. Rep..

[125] Li H.Y., Zhou D.D., Gan R.Y., Huang S.Y., Zhao C.N., Shang A., Xu X.Y., Li H.B. (2021). Effects and mechanisms of probiotics, prebiotics, synbiotics, and postbiotics on metabolic diseases targeting gut microbiota: A narrative review.. Nutrients.

[126] Garrett W.S., Lord G.M., Punit S., Lugo-Villarino G., Mazmanian S.K., Ito S., Glickman J.N., Glimcher L.H. (2007). Communicable ulcerative colitis induced by T-bet deficiency in the innate immune system.. Cell.

[127] Richards J.L., Yap Y.A., McLeod K.H., Mackay C.R., Mariño E. (2016). Dietary metabolites and the gut microbiota: An alternative approach to control inflammatory and autoimmune diseases.. Clin. Transl. Immunology.

[128] Sonnenburg E.D., Smits S.A., Tikhonov M., Higginbottom S.K., Wingreen N.S., Sonnenburg J.L. (2016). Diet-induced extinctions in the gut microbiota compound over generations.. Nature.

[129] Hua X., Zhu J., Yang T., Guo M., Li Q., Chen J., Li T. (2020). The gut microbiota and associated metabolites are altered in sleep disorder of children with autism spectrum disorders.. Front. Psychiatry.

[130] Agus A., Planchais J., Sokol H. (2018). Gut microbiota regulation of tryptophan metabolism in health and disease.. Cell Host Microbe.

[131] Ossenkopp K.P., Foley K.A., Gibson J., Fudge M.A., Kavaliers M., Cain D.P., MacFabe D.F. (2012). Systemic treatment with the enteric bacterial fermentation product, propionic acid, produces both conditioned taste avoidance and conditioned place avoidance in rats.. Behav. Brain Res..

[132] Hou Y., Li X., Liu C., Zhang M., Zhang X., Ge S., Zhao L. (2021). Neuroprotective effects of short-chain fatty acids in MPTP induced mice model of Parkinson’s disease.. Exp. Gerontol..

[133] Page M.J., Pretorius E. (2022). Platelet behavior contributes to neuropathologies: A focus on Alzheimer’s and Parkinson’s disease.. Semin. Thromb. Hemost..

[134] Abdel-Rahman E.A., Zaky E.A., Aboulsaoud M., Elhossiny R.M., Youssef W.Y., Mahmoud A.M., Ali S.S. (2021). Autism spectrum disorder (ASD)-associated mitochondrial deficits are revealed in children’s platelets but unimproved by hyperbaric oxygen therapy.. Free Radic. Res..

[135] Xie Z., Liu X., Huang X., Liu Q., Yang M., Huang D., Zhao P., Tian J., Wang X., Hou J. (2021). Remodelling of gut microbiota by Berberine attenuates trimethylamine N-oxide-induced platelet hyperreaction and thrombus formation.. Eur. J. Pharmacol..

[136] Anderson G., Rodriguez M., Reiter R.J. (2019). Multiple sclerosis: Melatonin, orexin, and ceramide interact with platelet activation coagulation factors and gut-microbiome-derived butyrate in the circadian dysregulation of mitochondria in glia and immune cells.. Int. J. Mol. Sci..

[137] Chen Z., Liu C., Jiang Y., Liu H., Shao L., Zhang K., Cheng D., Zhou Y., Chong W. (2020). HDAC inhibitor attenuated NETs formation induced by activated platelets in vitro, partially through downregulating platelet secretion.. Shock.

[138] Anderson G., Maes M. (2020). Gut dysbiosis dysregulates central and systemic homeostasis via suboptimal mitochondrial function: Assessment, treatment and classification implications.. Curr. Top. Med. Chem..

[139] Ghafouri-Fard S., Namvar A., Arsang-Jang S., Komaki A., Taheri M. (2020). Expression analysis of BDNF, BACE1, and their natural occurring antisenses in autistic patients.. J. Mol. Neurosci..

